# Diseases of the Rectum and Anus

**Published:** 1890-09

**Authors:** 


### Book Reviews.

DISEASES OF THE RECTUM AND ANUS, THEIR PATHOLOGY, DIAGNOSIS AND TREATMENT. By CHAS. B. KELSEY, A. B., M. D., Professor of Diseases of the Rectum at the New York Post-Graduate Medical School and Hospital; late Professor of Diseases of the Rectum at the University of Vermont, etc. Third edition; rewritten and enlarged, with two chromo-lithographs and one hundred and sixty-eight illustrations. New York: William Wood & Co. 1890. 8vo; pp. x.—483.

The first edition of Dr. Kelsey's book on Diseases of the Rectum and Anus was published in 1882, as one volume of Wood's Library of Standard Medical Authors. This book met, at that time, with a very cordial reception at the hands of the medical profession throughout the country. In 1884, the second edition of this work made its appearance as a separate volume, much enlarged, containing many valuable illustrations, and embodied in its text could be found all that was new in rectal pathology and treatment. Increasing experience in the study and practice of rectal surgery has prompted the author to give us a third edition of his valuable book. It is perhaps, today, the best American work that we have on the pathology and treatment of rectal disorders, and one well worthy the careful and studious attention of specialists and general practitioners of medicine. It is written in language both clear and concise, and ably presents the author's practical knowledge gained by a large clinical experience. Even a hurried perusal of the book will convince one that Prof. Kelsey is a patient worker and careful observer, keeping well to the fore in all that pertains to his chosen field of labor. As the author states in his preface:

The great advances which have been made during the past few years in the surgery of the rectum, and intestinal surgery generally, have necessitated many changes in this, the third edition of this book. The chapters on the treatment of strictures, both benign and malignant, and on the formation and closure of artificial anus, have therefore been entirely rewritten, and much new matter has been added.

Dr. Kelsey has spared neither time nor expense in the matter of illustrations, many of the cuts being made from photographs of his own cases taken by himself. The book is printed on good paper, in clear type, and is altogether a volume which reflects credit on both author and publishers. E. C.

